# Senolytic Properties of DR5-Selective TRAIL in Pancreatic Cancer Cell Lines

**DOI:** 10.32607/actanaturae.27844

**Published:** 2026

**Authors:** A. A. Isakova, N. V. Antipova, D. V. Mazur, E. I. Ivanova, D. A. Dolgikh, M. P. Kirpichnikov, M. E. Gasparian, A. V. Yagolovich

**Affiliations:** Faculty of Biology, Lomonosov Moscow State University, Moscow, 119234 Russia; Shemyakin–Ovchinnikov Institute of Bioorganic Chemistry, Russian Academy of Sciences, Moscow, 117997 Russia; National Research University Higher School of Economics, Moscow, 101000 Russia

**Keywords:** pancreatic adenocarcinoma, senescence, senolytic therapy, death receptor DR5, TRAIL, DR5-B, gemcitabine, doxorubicin

## Abstract

Pancreatic adenocarcinoma is one of the most aggressive cancers. Its treatment relies on conventional chemotherapy agents; particularly gemcitabine. Chemotherapy is known to induce cell cycle arrest and the development of a senescent phenotype in tumor cells. The accumulation of senescent cells limits tumor proliferation, followed by the secretion of factors of the senescence-associated secretory phenotype promoting malignancy in the tumor microenvironment and metastasis. This makes the search for drugs suitable for senolytic therapy highly relevant. This study explored the senolytic properties of a DR5 receptor-selective mutant variant of the antitumor cytokine TRAIL DR5-B in human pancreatic cancer cell lines, after prolonged co-incubation with gemcitabine or doxorubicin. In the MIA PaCa-2 and PANC-1 cell lines, both drugs significantly increased β-galactosidase activity and the expression of senescence markers, such as the cell cycle inhibitors p21 and p27; DR5-B effectively suppressed cell viability after chemotherapy treatment. In the BxPC-3 cell line, the drugs did not induce senescence and DR5-B cytotoxicity was virtually unchanged. Some features of senescence were observed in AsPC-1 cells; however, these cells remained resistant to DR5-B, presumably due to cFLIP overexpression. Hence, the DR5-B protein shows promise as a senolytic agent for the treatment of certain types of pancreatic adenocarcinoma.

## INTRODUCTION

Pancreatic adenocarcinoma (PDAC) is one of the most aggressive malignant neoplasms characterized by an extremely low patient survival. Only 20% of patients diagnosed with PDAC have a localized form that is amenable to surgical resection, in combination with preoperative or adjuvant chemotherapy, but the disease often relapses even after complete tumor resection [1]. Chemotherapy is the only treatment option available for most patients with inoperable cases or metastases [2]. The most commonly used regimens are gemcitabine and its combinations with other drugs, nab-paclitaxel and the FOLFIRINOX regimen (leucovorin, 5-fluorouracil, irinotecan and oxaliplatin); however, even modern treatment regimens provide only limited improvement in prognosis [3, 4]. Other approaches, including targeted therapy and immunotherapy, have not yet demonstrated significant efficacy in PDAC because of the molecular heterogeneity of the tumors and the immunosuppressive microenvironment [5].

In addition to the cytotoxic effect, many chemotherapeutics, including gemcitabine, induce a state of long-term cell cycle arrest in tumor cells known as senescence. Senescent cells are characterized by proliferation arrest, but they retain their metabolic activity and participate in immune processes. These cells exhibit a number of features, such as increased size and altered morphology, increased activity of the enzyme β-galactosidase (SA-β-gal), activation of the p53/p21 and p16 pathways, and production of factors of the senescence-associated secretory phenotype (SASP).

In non-pathological contexts, senescence serves as a protective mechanism to prevent tumorigenic transformation and other abnormalities [6]. Short-term exposure to SASP is known to promote immune surveillance and tissue regeneration, thereby limiting tumor cell proliferation, while chronic accumulation of senescent cells forms a pro-inflammatory microenvironment that supports tumor progression [7]. Importantly, SASP is considered a key pro-tumorigenic factor, because it promotes therapy resistance and metastasis through auto- and paracrine mechanisms by transforming neighboring cells (both tumor and non-tumor) and degrading the extracellular matrix [8, 9, 10]. Moreover, the presence of a dense stroma, which is particularly prominent in PDAC and impedes immune surveillance, exacerbates the accumulation of senescent cells and renders their contribution to disease progression especially significant [11]. This provides a rationale for developing senolytics to selectively eliminate SASP-positive cells and mitigate their negative impact on cancer progression.

In gemcitabine-resistant PDAC cell lines, gemcitabine was shown to induce a senescence phenotype, instead of apoptosis, which is accompanied by increased levels of the cell cycle inhibitors p21 and p19, PML (senescence-associated promyelocytic leukemia protein), and the DcR2 receptor; notably, senescence can be triggered even in the presence of p53 gene mutations [12]. In chemo-resistant PDAC cell lines, gemcitabine-induced senescent cells persist during treatment but the senolytic ABT-263 (navitoclax), which inhibits anti-apoptotic Bcl-2 family proteins, selectively destroys gemcitabine-induced senescent cells both in vitro and in tumor xenografts in mice in vivo. The combination of gemcitabine with ABT-263 was shown to significantly reduce tumor growth compared with monotherapy [13, 14]. These findings underscore the rationale for advancing senolytic strategies aimed at the efficient elimination of senescent cells. However, many of the known compounds with senolytic properties are highly toxic [15], which calls for the search for novel, effective molecules that exhibit a potent senolytic activity

In addition to Bcl-2 family inhibitors, senescent tumor cells are vulnerable to the activation of the exogenous apoptotic pathway via the TRAIL death receptor DR5. Selective DR5 receptor agonists have been shown to induce caspase-dependent apoptosis in chemotherapy-induced senescent tumor cells [16]. The resistance of senescent cells to DR5-mediated cell death is largely a result of the high levels of the anti-apoptotic protein cFLIP, a caspase-8 homolog. Inhibition of this mechanism, in combination with DR5 agonists, significantly enhances the senolytic effect both in vitro and in vivo [17].

The present study assesses the capacity of a modified receptor-selective cytokine TRAIL DR5-B (DR5 agonist) to trigger apoptosis in senescent PDAC cells induced by the exposure to the chemotherapeutic agents gemcitabine and doxorubicin.

## EXPERIMENTAL

**Cell lines and culture conditions**

PDAC cell lines (ATCC, USA) were maintained under standard culture conditions. AsPC-1 cells were grown in a RPMI-1640 medium (PanEco, Russia); and BxPC-3, MIA PaCa-2, and PANC-1 cells were grown in a DMEM medium (PanEco). The growth media contained fetal bovine serum (10%, Hyclone, USA), L-glutamine (1–2 mM, PanEco), and antibiotics penicillin (100 U/mL) and streptomycin (100 μg/mL). Cultivation was carried out at 37°C in an atmosphere containing 5% CO_2_ . A trypsin-EDTA solution (PanEco) was used to detach the cells from the culture plastic. Subcultures were performed every 3–4 days after the cells reached 70–80% confluence.

**Treating cells with drugs**

Gemcitabine (Tocris, UK) and doxorubicin (Tocris) were used to induce senescence. A receptor-selective mutant variant of the antitumor cytokine TRAIL DR5-B, obtained and purified previously, was used to evaluate the senolytic effect of the DR5 agonist [18, 19]. The cells were treated with chemotherapeutics at the following concentrations: AsPC-1 – 330 nM gemcitabine; BxPC-3, MIA PaCa-2, and PANC-1 – 33 nM gemcitabine; BxPC-3 – 10 nM doxorubicin; AsPC-1, MIA PaCa-2, and PANC-1 – 50 nM doxorubicin. Working concentrations of the chemotherapeutic agents were determined in preliminary titration experiments as doses that induce stable cell cycle arrest without massive cell death ( < 20%).

**Cell viability assessment**

Cell viability was determined using the WST-8 reagent (ServiceBio, China). After 7-day incubation with chemotherapy drugs, the cells were seeded into 96-well plates (10,000 cells/well) in a complete medium, and DR5-B (0–500 nM) was added 24 h later. Following a 72-h incubation period, the cells were incubated with WST-8 for 3 h and the optical density was measured at 450/595 nm. The data were normalized to the untreated control, which was set as 100%. The IC_50_ values were calculated in the GraphPad Prism 10.4.0 software using a three-parameter log(inhibitor) vs. response model.

**Assessment of SA-β-gal activity in the cells**

Senescent cells were detected after 7-day drug incubation using X-Gal substrate staining (1 mg/mL, TargetMol, USA), according to the standard protocol [20]. Cells were fixed with 2% paraformaldehyde, incubated at 37°C for 24–48 h until a blue stain had developed, and images were captured using an Eclipse TS100-F inversion microscope (Nikon, Japan). The staining intensity was evaluated using the ImageJ software (NIH, USA) by measuring the integrated signal density with normalization to the area and background correction.

**Quantitative reverse transcription PCR (RT-qPCR)**

Total RNA was isolated using the ExtractRNA reagent (Evrogen, Russia), and the concentration was determined using a NanoDrop One C spectrophotometer (Thermo Fisher Scientific, USA). cDNA synthesis was performed using an MMLV RT kit (Evrogen, Russia), according to the manufacturer’s protocol. Real-time PCR was performed using a LightCycler 96 instrument (Roche, Switzerland) with the qPCRmix-HS SYBR reagent (Evrogen) under standard conditions. The absence of nonspecific products was monitored via melting curves. 18S rRNA was used as the internal control. Relative gene expression levels were calculated using the 2–ΔΔCt method; differences were considered significant at p < 0.05. Primers for detecting p21Waf1, p27KIP1, and cFLIP expression are listed in Table 1.

**Table 1:** Primers for RT-qPCR

No.	Gene	Sequence (5'-3')	
		Forward	Forward
1	p21 (CDKN1A-F110)	AGTCAGTTCCTTGTGGAGCC	CATTAGCGCATCACAGTCGC
2	p27 (CDKN1B-F115)	TGTTTCAGACGGTTCCCCAAA	CCATTCCATGAAGTCAGCGAT
3	cFLIP (CFLAR)	GGCTCCCCCTGCATCACATC	CCGCAGTACACAGGCTCCAGA

**Flow cytometry**

Surface expression of the DR5 receptor was detected by flow cytometry. After 7-day chemotherapy treatment, cells were detached with an EDTA solution, incubated for 1 h at 4°C with anti-DR5 monoclonal antibody (clone DR5-01-1) (GeneTex, USA), washed three times with a FACS buffer, and incubated for another 1 h at 4°C with secondary antibodies DyLight 488 (GeneTex, USA). Isotype control with mouse IgG1 was stained in parallel. Detection was performed using a CytoFLEX flow cytometer (Beckman Coulter, USA), and DR5 levels were expressed as ΔMFI relative to the isotype control.

**Analysis of cell growth dynamics**

Cell counts using a Goryaev chamber were performed to assess the cell growth dynamics following 7-day incubation with the chemotherapeutics. Viable cells were identified by trypan blue exclusion. The growth inhibition index (GI) was calculated using the formula:

GI (%) = (N_sample_ − N_0_ ) / (N_control_ − N_0_ ) × 100.

The GI values were interpreted as follows: GI = 100% corresponds to growth equivalent to the control; GI ≈ 0% is complete proliferation arrest at the seeding level; and GI < 0% corresponds to cytotoxicity.

**Transcriptomic data analysis**

RNA-seq data for the pancreatic cell lines were obtained from the DepMap (Omics Expression ProteinCoding Genes, normalized log2 (TPM+1)) database [21, 22]. Expression of the genes of interest in the studied cell lines was analyzed. Data processing and visualization were performed in the R environment version 4.3.2 (R Foundation for Statistical Computing, Austria; https://www.r-project.org/) using the ggplot2 package (version 3.4.4).

**Statistical analysis**

Data were statistically processed using the GraphPad Prism 10.4.0 software. Experiments were performed in triplicates; results are presented as mean ± SEM. Differences were assessed by ANOVA (or Welch’s ANOVA in case of unequal variances) with Dunnett’s test; p < 0.05 was considered significant.

## RESULTS AND DISCUSSION

**Senescence induction in PDAC cell lines by gemcitabine and doxorubicin**

PDAC is characterized by pronounced genetic and phenotypic heterogeneity, which is evident in the variability observed across different cellular models in vitro [23]. Previous studies have shown that PDAC cell lines respond differently to chemotherapeutics and exhibit varying capacities for developing a senescent phenotype [13, 14, 24]. In our experiments, four lines of human PDAC were studied: MIA PaCa-2, PANC-1, BxPC-3, and AsPC-1. To induce senescence, gemcitabine was employed as a drug clinically used for PDAC treatment, along with doxorubicin, a well-established model chemotherapeutic agent due to its ability to cause DNA damage and trigger senescence [25, 26]. Both drugs were used at subtoxic concentrations selected in preliminary experiments (up to 20% cell death). Cellular senescence was confirmed by several established criteria: assessment of the cell growth rate, staining for SA-β-gal enzyme activity, and relative expression of p21 and p27 [27].

**Fig. 1:**
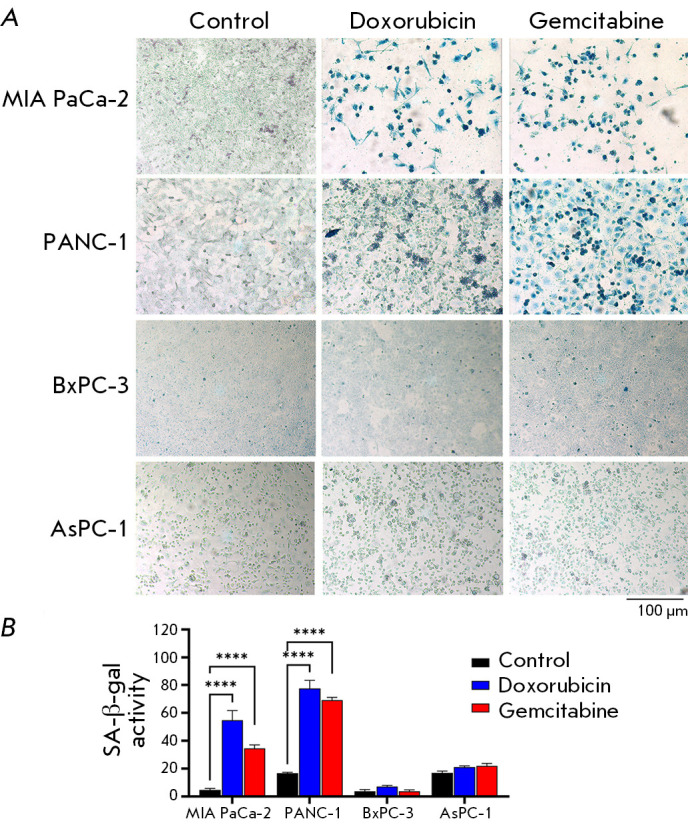
SA-β-gal activation in PDAC cell lines MIA PaCa-2, PANC-1, BxPC-1, and AsPC-1 following 7-day incubation with doxorubicin and gemcitabine at subtoxic doses. (A) Cell staining for SA-β-gal activity. Scale bar: 100 μm. (B) Quantitative analysis of staining intensity (mean ± SEM, n = 3, ****p < 0.0001)

The investigated cell lines exhibited varying susceptibility to senescence induction. SA-β-gal staining revealed a marked accumulation of positively stained cells in MIA PaCa-2 and PANC-1, whereas the BxPC-3 and AsPC-1 lines showed no obvious staining (Fig. 1A). Quantitative analysis of staining intensity using the ImageJ software confirms these observations (Fig. 1B). Staining intensity in MIA PaCa-2 cells increased 7- and 11-fold after exposure to gemcitabine and doxorubicin, respectively; in PANC-1 cells, it increased 4- and 5-fold. No significant changes in SA-β-gal activity staining were detected in the AsPC-1 and BxPC-3 cell lines (Fig. 1B).

**Fig. 2:**
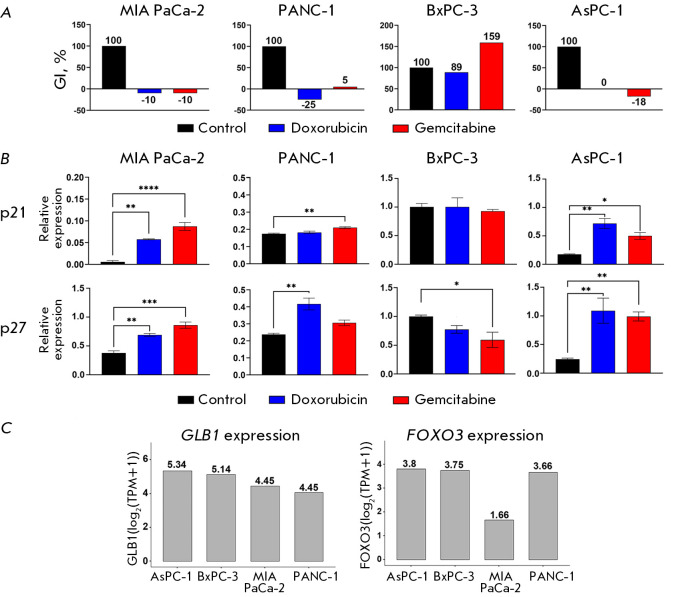
Molecular and functional markers of senescence in PDAC cell lines treated with doxorubicin or gemcitabine. (A) Growth inhibition index (GI) after 7-day incubation with chemotherapeutics. (B) Relative expression of the CDKN1A (p21) and CDKN1B (p27) genes by RT-qPCR (mean ± SEM, n = 3, *p < 0.05, **p < 0.01, ***p < 0.001, ****p < 0.0001). (C) Expression of the GLB1 and FOXO3 genes in PDAC cell lines (log2 (TPM+1)), derived from the RNA-seq data in the DepMap database

Since proliferation arrest is the hallmark of senescent cells, this parameter was quantified using the cell growth index (GI). The obtained GI values indicated pronounced inhibition of proliferation in the MIA PaCa-2, PANC-1, and AsPC-1 cells with minimal cytotoxic effects (MIA PaCa-2: -10% upon treatment with doxorubicin and gemcitabine; PANC-1: -25% for doxorubicin, 5% for gemcitabine; AsPC-1: 0% and -18%, respectively) (Fig. 2A). Doxorubicin failed to significantly suppress the proliferation of BxPC-3 cells (Fig. 2A), whereas gemcitabine increased GI by 159%, which may be attributed to the adaptive proliferative mechanisms under subtoxic stress conditions. Similar growth following gemcitabine treatment was observed in previous studies, where the drug at low doses stimulated metastasis in the BxPC-3 xenograft model in vivo [28]. This phenomenon is consistent with the concept of chemotherapeutic hormesis, which posits that subtoxic doses of cytostatics may induce tumor growth stimulation and progression, rather than suppression [29].

The upregulation of the cyclin-dependent kinase inhibitors p21 and p27 attests to activation of the mechanisms underlying permanent cell cycle arrest, thereby confirming the establishment of a senescent phenotype in tumor cells [30, 31]. According to the RT-qPCR data, gemcitabine and doxorubicin induced a significant upregulation of p21/p27 expression in MIA PaCa-2 and AsPC-1, whereas in PANC-1, the p21 level increased only upon treatment with gemcitabine, while p27 rose solely after exposure to doxorubicin (Fig. 2B). No significant changes were observed in the BxPC-3 cells, and p27 levels even decreased slightly after gemcitabine treatment. It is known that the induction of p21 and p27 expression during senescence is largely determined by the TP53 (p53) gene status, and PDAC cell lines exhibit marked heterogeneity in this regard. The induction of p21 and p27 varied across the cell lines and did no directly depend on the p53 status. Discordant marker elevation or no changes were observed in PANC-1 and BxPC-3 cells, which is consistent with the partially defective transcriptional activity of mutant p53 in these cells [32]. In MIA PaCa-2 and AsPC-1, regardless of mutations or the absence of p53, chemotherapy induced a pronounced upregulation of p21 and p27, which likely indicates the activation of the p53-independent senescence pathways (e.g., via p73/TAp63 or TGF-β/SMAD) [33, 34].

In AsPC-1 cells, despite the increase in p21/p27 and the decrease in GI (Fig. 2A,B), SA-β-gal activity remained unchanged (Fig. 2A). According to the data from the DepMap database, a similar discrepancy may be attributed to a high basal expression level of the β-galactosidase gene GLB1, as evidenced by the DepMap data (Fig. 2B) or to the limited capacity of the cells to increase their lysosomal mass, which determines SA-β-gal activity

FOXO3 (a transcription factor of the FOXO family) is known to be among the key factors that maintain cellular resilience and prevent senescence by regulating the expression of the stress response genes [35]. Recent studies have shown that increased FOXO3 activity confers cellular stress resistance, reduces SA-βGal activity, restricts SASP, and promotes tissue homeostasis maintenance [36]. According to the DepMap data, FOXO3 expression was minimal in MIA PaCa-2 and high in AsPC-1, BxPC-3, and PANC-1 (Fig. 2C). The development of a classical senescent phenotype in MIA PaCa-2 may be associated with a deficiency of FOXO3-mediated protective mechanisms [35].

Thus, chemotherapy induced senescence in three of the four lines (MIA PaCa-2, PANC-1, AsPC-1), which was most pronounced in MIA PaCa-2.

**Senolytic activity of the DR5-selective cytokine TRAIL DR5-B in PDAC cell lines with chemotherapy-induced senescence**

**Fig. 3:**
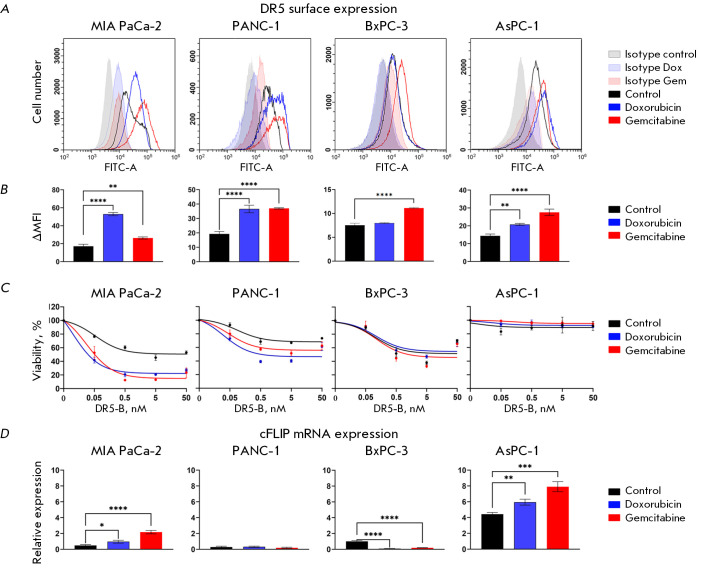
Cytotoxicity of DR5-B in PDAC cell lines after pre-incubation with chemotherapeutics. (A) DR5 receptor expression in PDAC cell lines after 7-day incubation with doxorubicin or gemcitabine, flow cytometry histograms (FITC-A). (B) The ΔMFI values (anti-DR5 – isotype) (mean ± SEM, n = 3, *p < 0.05, **p < 0.01, ***p < 0.001, ****p < 0.0001). (C) Viability of tumor cells after 72-h incubation with DR5-B. (D) cFLIP expression measured by RT-qPCR (mean ± SEM, n = 3, *p < 0.05, **p < 0.01, ***p < 0.001, ****p < 0.0001)

Previously, we generated a receptor-selective mutant variant of the antitumor cytokine TRAIL DR5-B, which binds selectively to the death receptor DR5 [18]. DR5-B exhibited an enhanced cytotoxic activity against various tumor cell lines both in vitro and in vivo, which was attributed to its lack of affinity for the TRAIL decoy receptors DcR1 and DcR2, whose upregulation is associated with senescence [37, 38]. To assess the senolytic potential of DR5-B, its cytotoxicity was evaluated in PDAC cell lines following pre-incubation with doxorubicin or gemcitabine. Chemotherapeutics are known to upregulate DR5 receptor expression in tumor cells [39, 40]. After 7-day exposure to gemcitabine or doxorubicin, a significant increase in surface DR5 receptor expression was observed in MIA PaCa-2 cells (225% after gemcitabine and 64% after doxorubicin), PANC-1 (87% after gemcitabine and 83% after doxorubicin), and AsPC-1 (92% after gemcitabine and 41% after doxorubicin) (Fig. 3A,B). In BxPC-3 cells, a significant increase in surface DR5 expression (46%) was detected only after treatment with gemcitabine.

**Table 2:** The IC50 values of DR5-B in PDAC cell lines after 7-day pre-treatment with chemotherapeutics

Cell line	Control	Gemcitabine	Doxorubicin
PANC-1	0.085 ± 0.037	0.044 ± 0.018	0.031 ± 0.019
MIA PaCa-2	0.054 ± 0.015	0.027 ± 0.008	0.0096 ± 0.0028
BxPC-3	0.102 ± 0.067	0.113 ± 0.074	0.109 ± 0.051
AsPC-1	n.d.	n.d.	n.d.

Consequently, MIA PaCa-2 cells showed pronounced sensitization to DR5-B, with a substantial decrease in the drug’s IC_50_: 1.9-fold after gemcitabine treatment and 5-fold after doxorubicin exposure (Fig. 3B, Table 2). Pre-incubation with chemotherapeutics also enhanced the sensitivity of PANC-1 cells to DR5-B: the IC_50_ value of DR5-B was 1.8-fold and 2.7-fold lower after treatment with gemcitabine and doxorubicin, respectively. The sensitivity of BxPC-3 cells to DR5-B remained virtually unchanged after incubation with chemotherapeutics. Notably, the phenotype of these cells did not meet any of the criteria for a senescent phenotype.

The senolytic effect of DR5-B was completely absent in DR5-B-resistant AsPC-1 cells (Fig. 3B), which is likely due to the high levels of the anti-apoptotic protein cFLIP, whose expression increased following chemotherapy treatment (Fig. 3D). The level of the cell death suppressor cFLIP is known to vary across PDAC cell lines, and its upregulation is associated with resistance to DR5-dependent apoptosis [41, 42]. Our findings reveal that the cFLIP expression level was highest in AsPC-1, decreased in BxPC-3, and did not affect the sensitivity of MIA PaCa-2 and PANC-1. Therefore, a combination strategy may be promising, in which cFLIP inhibition enhances the sensitivity of senescent cells to DR5 activation, as previously demonstrated in several models [17].

Our findings indicate that the selective DR5 receptor agonist, the DR5-B protein, exhibits a senolytic effect in cells developing a mature senescent phenotype, whereas cell lines retaining proliferative capacity or characterized by high levels of anti-apoptotic regulators (such as cFLIP) remain resistant. These data emphasize the importance of considering tumor clonal heterogeneity when developing novel therapeutic approaches. Notably, targeting the DR5 receptor, in combination with suppression of the key anti-apoptotic factors, can be a promising strategy for selective elimination of chemotherapy-induced senescent PDAC cells.

## CONCLUSION

Research conducted over recent decades has shown that senolytic drugs represent an exceptionally promising direction in the development of novel methods of antitumor treatment, such as senolytic therapy [43]. The so-called “one-two punch” approach is a popular strategy: in it, cancer cells first become vulnerable due to chemotherapy-induced senescence and are then eliminated by senolytic agents. Senolytic therapy has a potential to prevent tumor metastasis, treatment resistance, and relapse by selectively eradicating senescent tumor cells. The field is advancing rapidly, accompanied by a surge in preclinical and clinical trials. These studies will shape the future of senolytic therapy by assessing its safety and efficacy for patients. Our study demonstrates for the first time the senolytic activity of a DR5 death receptor agonist, specifically a receptor-selective variant of the antitumor cytokine, TRAIL DR5-B, in pancreatic adenocarcinoma cell lines following treatment with the chemotherapeutic agents doxorubicin and gemcitabine. Targeting the DR5 receptor can be a promising strategy for selectively eliminating senescent tumor cells, particularly when combined with suppression of the key factors mediating cellular resistance to apoptosis. Preclinical trials in animal models will be needed to assess the therapeutic potential of DR5-B as a senolytic agent.

## Abbreviations

GI: growth inhibition index
SASP: senescence-associated secretory phenotype
TRAIL: tumor necrosis factor (TNF)-related apoptosis-inducing ligand
PDAC: pancreatic ductal adenocarcinoma
